# Open-Access Worldwide Population STR Database Constructed Using High-Coverage Massively Parallel Sequencing Data Obtained from the 1000 Genomes Project

**DOI:** 10.3390/genes13122205

**Published:** 2022-11-24

**Authors:** Tamara Soledad Frontanilla, Guilherme Valle-Silva, Jesus Ayala, Celso Teixeira Mendes-Junior

**Affiliations:** 1 Departamento de Genética, Faculdade de Medicina de Ribeirão Preto, Universidade de São Paulo, Ribeirão Preto 14049-900, SP, Brazil; 2 Departamento de Química, Laboratório de Pesquisas Forenses e Genômicas, Faculdade de Filosofia, Ciências e Letras de Ribeirão Preto, Universidade de São Paulo, Ribeirão Preto 14040-901, SP, Brazil; 3 Facultad de Ingeniería Informática, Universidad de la Integración de las Americas, Asunción 00120-6, Paraguay

**Keywords:** HipSTR, allele frequencies, forensic genetics, worldwide population, bioinformatics

## Abstract

Achieving accurate STR genotyping by using next-generation sequencing data has been challenging. To provide the forensic genetics community with a reliable open-access STR database, we conducted a comprehensive genotyping analysis of a set of STRs of broad forensic interest obtained from 1000 Genome populations. We analyzed 22 STR markers using files of the high-coverage dataset of Phase 3 of the 1000 Genomes Project. We used HipSTR to call genotypes from 2504 samples obtained from 26 populations. We were not able to detect the D21S11 marker. The Hardy-Weinberg equilibrium analysis coupled with a comprehensive analysis of allele frequencies revealed that HipSTR was not able to identify longer alleles, which resulted in heterozygote deficiency. Nevertheless, AMOVA, a clustering analysis that uses STRUCTURE, and a Principal Coordinates Analysis showed a clear-cut separation between the four major ancestries sampled by the 1000 Genomes Consortium. Except for larger Penta D and Penta E alleles, and two very small Penta D alleles (2.2 and 3.2) usually observed in African populations, our analyses revealed that allele frequencies and genotypes offered as an open-access database are consistent and reliable.

## 1. Introduction

Next-generation sequencing (NGS), also known as massively parallel or deep sequencing, is a technology that allows millions of DNA fragments to be sequenced in parallel. NGS can deal with several regions or targets simultaneously, enabling variation sites or mutations in the genome to be detected. This technology has allowed worldwide human genetic diversity to be studied for various purposes, including forensic human identification [1,2,3].

Advances in the genomics area have made it possible to use NGS techniques in a more accessible way, mostly because of lower costs. Currently, many researchers are performing whole-exome (WES) and even whole-genome (WGS) sequencing to estimate polygenic risk scores and probabilities of developing multifactorial diseases associated with various genetic regions at once, which would be a more laborious and costly issue if using traditional methodologies [1].

The 1000 Genomes Consortium is a worldwide collaboration that has produced an extensive catalog of human genetic variation. The consortium has sequenced whole genomes of 2504 individuals belonging to multiple populations derived from five population groups: African, East Asian, European, South Asian, and admixed Americans [4]. These data are freely available at the International Genome Sample Resource website (https://www.internationalgenome.org; accessed on 5 July 2021) to generate a variant call format file that uses a set of specific command lines [5]. In 2015, during Phase 3 of the Project, the consortium analyzed the genomes of all the individuals by using a combination of low-coverage whole-genome sequencing (WGS), deep exome sequencing, and dense microarray genotyping. The consortium described worldwide patterns of genomic diversity on the basis of Single Nucleotide Polymorphisms (SNPs), indels, and structural variants (SVs), including deletions, insertions, duplications, inversions, and copy-number variants (CNVs), but it did not analyze or study short tandem repeat (STR) markers in depth [6].

STR markers are crucial in human identification. These markers have high polymorphism levels and are particularly useful for interpreting mixtures of biological samples. However, in addition to the issue of small-sized amplicons, genotyping STR markers by using NGS data is difficult because alignment and stutter errors are frequent [7]. Achieving accurate genotyping by employing NGS data has been challenging because these data have high sequencing error rates [8]. Gymrek et al. (2012) managed to obtain and to analyze STR markers from the dataset of the 1000 Genomes Project using lobSTR [9]. Given that high coverage is mandatory for reliable STR genotype calling to be achieved, a primary concern regarding that study was that the data obtained from the 1000 Genomes Project available for lobSTR were generated by employing shallow sequencing coverage (2x–6x), so the calling was potentially susceptible to errors [10].

To circumvent this coverage issue, the New York Genome Center (NYGC) recently re-sequenced the 2504 samples of the panel of Phase 3 of the 1000 Genomes Project with high (30x) coverage, and aligned the sequence data to GRCh38. These publicly available data could be used to call STR markers reliably [5,11].

NGS technology allows dozens of STR markers to be analyzed together with different classes of markers that provide complementary contributions to population genetics and human identification. For example, including SNPs used as predictors of ancestry and phenotypic characteristics into commercial kits that employ capillary electrophoresis is unfeasible, but they can be combined with STR markers in NGS assays [1]. The problem with the NGS technology is the large amount of data generated and the lack of bioinformatic tools to analyze it [1]. Some tools (e.g., lobSTR [9], STRait Razor [12], toaSTR [13], and HipSTR [10], among others) were developed to analyze STR markers by using NGS data. Each tool employs different algorithms and flanking regions to capture STR reads.

Haplotype inference and phasing for STRs (HipSTR) was developed for calling microsatellites specifically from WGS Illumina FASTq files. HipSTR was designed to deal with genotyping errors and to obtain more robust STR genotypes. HipSTR accomplished this by learning locus-specific PCR stutter models, with the aid of an EM algorithm, by employing a specialized hidden Markov model to align reads to candidate alleles while accounting for STR artifacts, and by using phased SNP haplotypes to genotype and to phase STR markers. These factors turned HipSTR into one of the most reliable tools for genotyping STRs from Illumina sequencing data [10,14].

In contrast to other tools, HipSTR can process hundreds of samples at once. It also allows the user to determine the set of STR markers that must be analyzed and the flanking regions that must be used to capture them. In fact, previous studies showed that HipSTR provides accurate genotype calling. HipSTR accuracy was tested by comparing WGS calls from 118 samples to capillary electrophoresis data, which resulted in 98.8% consistency [10,15]. Recently, we compared HipSTR with Strait Razor and toaSTR, to find that the three tools present high allele calling accuracy (greater than 97%) [14]. Although data processing with HipSTR is more complex and requires bioinformatics knowledge and some nomenclature adjustments, this tool is currently the fastest and most appropriate to deal with larger datasets, including whole genomes [14].

In this investigation we conducted a comprehensive genotyping analysis of a set of STRs of broad forensic interest obtained from the 1000 Genomes populations, aiming to release a reliable open-access STR database that should contribute to future studies in the field of forensic genetics.

## 2. Materials and Methods

### 2.1. Genotype Calling

Genotypes were called from 2504 individuals belonging to 26 populations derived from five population groups analyzed by the 1000 Genomes Consortium, namely African (AFR), East Asian (EAS), European (EUR), South Asian (SAS), and admixed American (AMR) [4]. The NYGC re-sequenced the samples of Phase 3 of the 1000 Genomes Project in a high-coverage (30x) assay by applying the NovaSeq 6000 Sequencing System (Illumina, Inc.; San Diego, CA, USA) with a paired-end approach (2 × 150 bp). Then, the NYGC made the data freely available at https://www.internationalgenome.org/data-portal/data-collection/30x-grch38 (accessed on 10 July 2021).

We used CRAM files to obtain the STR genotypes with the aid of the HipSTR software [10]. We selected 22 autosomal microsatellites that are commonly used in forensic practice: CSF1PO, D1S1656, D2S441, D2S1338, D3S1358, D5S818, D7S820, D8S1179, D10S1248, D12S391, D13S317, D16S539, D18S51, D19S433, D21S11, D22S1045, FGA, Penta D, Penta E, TH01, TPOX, and vWA.

To genotype the 22 STR markers based on the human reference genome GRCh38, we ran the HipSTR algorithm for each individual. For this purpose, we used a BED file with the coordinates of each STR region of interest, which was available in the HipSTR repository [10] (https://hipstr-tool.github.io/HipSTR-tutorial/; accessed on 10 July 2021) as described elsewhere [14]. We applied the calling filter (15% stutter model) and a minimum of eight reads to obtain more reliable genotypes. According to a binomial distribution, this minimum number of reads ensures (*p* > 0.99) that a homozygous genotype is called because of lack of variability at a given locus and not because the second allele has not been sampled.

To perform genotype calling, we used the VCF output file produced by HipSTR and took three parameters into account: the reference allele of each marker, the period (i.e., the length of each STR repeat unit), and the base pair differences (GB) as compared to the reference allele. We adjusted the nomenclature for D19S433, Penta D, Penta E, and vWA by following the recommendations made by Valle-Silva et al. [14]: removal of two repeat units from all D19S433 and vWA alleles called by HipSTR, inclusion of one repeat unit into all Penta D alleles, and removal of two nucleotides from all Penta E alleles. By using IGV software 2.8.2 [16,17] and the HipSTR VizAln function [10], Valle-Silva et al. [14] demonstrated that such adjustments are necessary to prevent some base pairs from shifting in allele calling when compared to the nomenclature established by the ISFG [18].

### 2.2. Statistical Analysis

We calculated allele frequencies, the Hardy-Weinberg equilibrium, and forensic parameters {Match Probability (MP), Power of Discrimination (PD), Power of Exclusion (PE), and Polymorphism Information Content (PIC)} for each population sample or each population group using GenAlEx 6.5 [19] and STRAF 2.5.1 [20] software.

We employed Principal Coordinates Analysis (PCoA) using GenAlEx [19], Analysis of Molecular Variance (AMOVA) using Arlequin [21], and clustering analyses using STRUCTURE 2.3.4 [22] to explore how genetic diversity is distributed across populations of different ethnic backgrounds. We performed STRUCTURE analysis for *k* ranging from 3 to 6 by applying the correlated allele frequencies model, 100,000 burn-in steps followed by 100,000 Markov Chain Monte Carlo interactions, in 100 independent runs. We selected the results from the runs with the largest “Estimated Ln Probability of Data” {LnP (D)} and depicted them in bar plots created with Distruct 1.1 [23].

We also compared the allele frequencies estimated from the 1000 Genomes Project dataset with STR data retrieved from the same five major population groups (African, European, East Asian, South Asian, and admixed American) that compose the SPSmart STR browser (PopSTR) [24]. For this purpose, we employed Arlequin software to compare the allele frequencies of each STR marker for a given population group between the two datasets by using *F_ST_* and an exact test of population differentiation based on genotype frequencies [21]. We made this comparison to verify the reliability of genotype data generated by HipSTR.

## 3. Results

The STR genotypes defined for each individual from the newest dataset released by the 1000 Genomes Project are available in Supplementary Table S1 as an open-access database. We excluded the D21S11 marker because we did not succeed in genotyping it (See discussion). Apart from this marker, the mean coverage for calling genotypes ranged from 37.14 (TPOX) to 52.53 (D12S391) (Table 1). The average successful calling rate was 98.59%; this rate ranged from 84.18% (Penta E) to 100% (CSF1PO, D2S441, D2S1338, D3S1358, D5S818, D8S1179, D22S1045, and TPOX) (Table 2).

Table 2 lists the allele frequencies and forensic parameters estimated for the whole dataset. The allele frequencies and forensic parameters estimated for each of the 26 populations (Supplementary Table S2) and the five population groups (Supplementary Table S3) are available as Supplementary Data. In general, the most polymorphic loci in all the populations were D1S1656, D2S1338, D12S391, D18S51, and FGA (Table 2). The analyzed loci were highly informative, with elevated PD values ranging between 86.59% (TPOX) and 97.76% (D1S1656). The combined MP was 5.72 × 10^−27^, and the combined PE was 0.99999997. Analysis of each locus in each population (Supplementary Table S2) showed that D22S1045 in PEL (71.61%) and D1S1656 in GBR (97.52%) presented the lowest and the highest PD value, respectively. The combined MP ranged from 1.98 × 10^−25^ in ACB to 2.20 × 10^−21^ in PEL.

We estimated the adherences of genotype frequencies to Hardy-Weinberg Equilibrium expectations for each STR marker at a population level (Table 3). Penta E presented heterozygote deficiency in 24 out of the 26 populations, leading to departures from the Hardy-Weinberg equilibrium. This finding indicated that HipSTR incorrectly called many heterozygous genotypes as homozygous. Disregarding Penta E, the number of deviations ranged from one (D13S317 and D16S539) to five (D19S433 and Penta D), and the number of deviations across populations ranged from zero (ASW and CEU) to seven (PUR), with an average of 2.42 departures in each population. When we considered the Bonferroni correction for multiple tests, only 39 departures remained significant, and most of them (61.53%) concerned Penta E.

**Figure 1:**
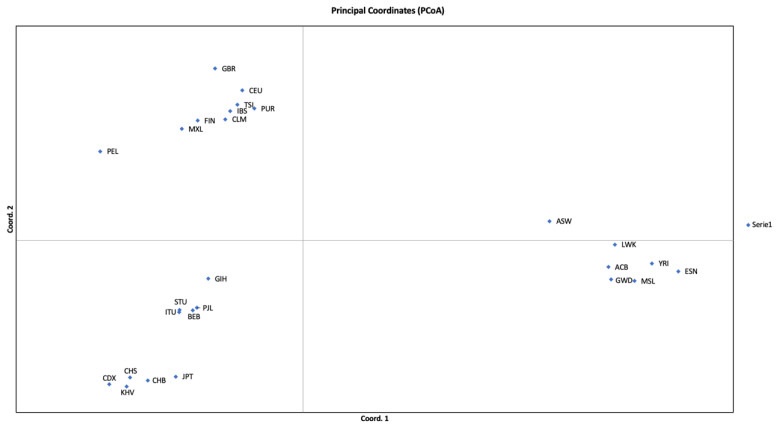
Principal Coordinates Analysis (PCoA) based on autosomal STR data regarding the 26 populations analyzed in the 1000 Genomes Project. Each point represents a population sample. More details on these populations are available in Supplementary Table S2. Coordinates 1 and 2 account for 39.15% and 19.25% of the variance, respectively.

Principal Coordinates Analysis (PCoA) revealed four different population clusters (Figure 1). The first coordinate separated the cluster of African (AFR) populations on the right side. On the left side, we observed three different population groups: the European (EUR) populations in the upper part, the East Asian (EAS) populations in the lower section, and the South Asian (SAS) populations between them. The CLM, MXL, PEL, and PUR admixed populations clustered with the European populations, while the ACB and ASW populations clustered with the African (AFR) populations, reflecting their ancestry compositions.

**Figure 2:**
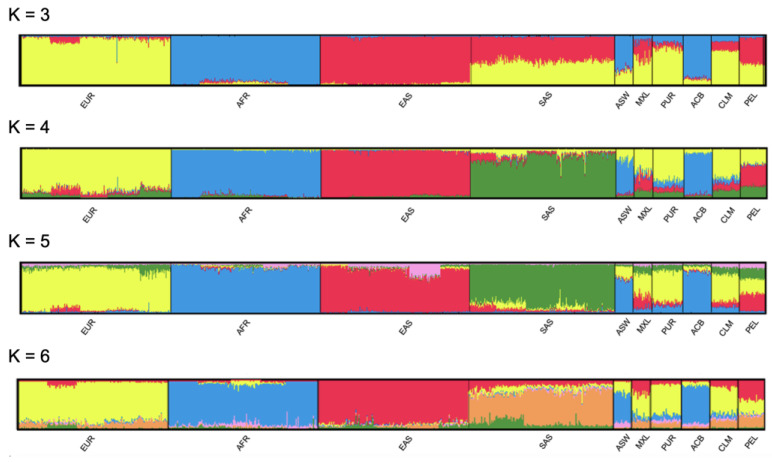
STRUCTURE analysis based on autosomal STR data obtained from the 26 populations included in the 1000 Genomes Project. Five sets of 100 independent runs, with the number of clusters ranging from 3 to 6, were conducted. Each bar plot depicts the results obtained from the run with the largest LnP (D) for the given *k*.

We obtained similar results when we conducted the STRUCTURE analysis. Figure 2 depicts the STRUCTURE results derived from runs obtained with *k* ranging from three to six. When *k* = 4, each cluster reflected one of the major ancestries of the 1000 Genomes Project. Moreover, each of the six admixed American populations presented varying levels of ancestries from the four biogeographical groups. To verify the distribution of variance in different levels, we performed AMOVA by assuming a hierarchical structure that gathered the populations in four population groups: AFR, EAS, EUR, and SAS. We did not take the six populations in the AMR population group into account because their admixed compositions would bias the AMOVA results by reducing the proportion of variance between groups. We observed most of the variance within populations (97.12%). Differences between the four population groups accounted for 2.54% of the variance, whereas only 0.34% of the variance occurred due to differences between populations belonging to the same group.

By using *F_ST_*, we also compared the allele frequencies estimated from the dataset of the 1000 Genomes Project to the STR data retrieved for the same five major population groups (African, European, East Asian, South Asian, and admixed American) that composed the SPSmart STR browser (PopSTR) [24] (Table 4). While the AMR (four), EAS (three), EUR (eight), and SAS (four) population groups presented small numbers of markers with significantly different frequencies between the two datasets, AFR presented 17 significant differences. This pattern might reflect the set of populations that compose the compared groups. Penta E was the only marker that showed significantly different *F_ST_* values in all comparisons. By leaving AFR and Penta E aside, we observed only 15 significant differences out of 80 comparisons: the mean number of statistically significant differences was 0.75 per marker; this number ranged from zero (eight STR markers) to three (D2S441). When we considered the Bonferroni correction for multiple tests, only three of these 15 *F_ST_* values remained significant, while six out of 16 significant differences observed for AFR (leaving Penta E aside), and all five Penta E differences remained significantly different.

## 4. Discussion

The present study provides the most diverse database of forensic autosomal STR markers obtained from global populations. STR markers display high levels of polymorphism, which makes them attractive for forensic purposes and population genetics studies. This is the first time that the 1000 Genomes high-coverage (~30x) dataset has been used for STR genotyping purpose. Although a few previous initiatives [9,25,26] attempted to genotype forensically relevant STRs, they only dealt with previous low-coverage 1000 Genomes releases (~7.4x), which prevented the acquisition of results or resulted in highly unreliable genotypes due to large rates of allele dropout. Moreover, it should be emphasized that even the last paper that presented the high-coverage WGS data did not include STR variants in the results and stated that genotyping STRs from such data remains a considerable challenge [27].

In forensic genetics, STR markers consist in the most widespread and informative tool for human identification. In spite of the limitations addressed below, such as unreliability of Penta D and Penta E genotypes involving specific alleles, this NGS-based STR database presents reliable allele frequencies that could be used in criminal casework to estimate the rarity of a given STR-based profile from a query sample of unknown or uncertain ancestry in various worldwide populations. This could instantly, and without additional costs, trigger a DNA-based intelligence strategy to guide enquiries [28] providing hints and/or assigning biogeographical origin in many situations, such as a missing person investigation [28,29], leaving only the most complex cases for supplementary analysis with a most suitable set of Ancestry Informative Markers.

Short-read next generation sequencing is slowly being introduced in forensic labs worldwide. Although such technology is still restricted and expensive, it has become more sensitive, requiring as little as 25 pg of extracted DNA, and is suitable to solve more complex cases, such as discrimination of twins (using STRs, WGS or mtDNA sequencing approaches) and deconvolution of highly unbalanced mixtures reviewed by [30]. Some criminal [31,32,33], kinship [34] and missing persons [35] casework already benefiting from this have been reported. However, genotyping STR markers by using NGS data, especially WGS assays, may be challenging—accurate genotyping requires high coverage, longer alleles are difficult to detect due to reads of limited sizes, and mutations in flanking regions may lead to null alleles [36]. These and other issues have been addressed by Gaag et al. [37] and Valle-Silva et al. [14].

Notwithstanding the challenges addressed here, several studies have demonstrated that STRs can be genotyped by using dedicated bioinformatics tools. Software such as LobSTR [9], toaSTR [13], STRait Razor [12], and HipSTR [10], among others, have shown consistent and accurate results [14,15]. Moreover, Bornman et al. [8] demonstrated that, by using an NGS approach, CODIS loci could be accurately called even from mixtures.

Particularly for the deconvolution of mixtures, the identification of isometric alleles (i.e., alleles with the same length but containing different repeat sequences) is a necessary task, since it further increases the discriminating power of the currently used STR markers; nevertheless, it is not achieved with traditional PCR and capillary electrophoresis techniques [2,3]. This sequence-based analysis is already feasible with small-scale targeted sequencing assays, particularly those using kits and software solutions tailored for forensic purposes, such as the ForenSeq DNA Signature Prep Kit coupled with the ForenSeq™ Universal Analysis Software (Verogen Inc., San Diego, CA, USA) [38] or the Precision ID GlobalFiler™ NGS STR Panel v2 coupled with the Converge Software NGS Analysis Module (Thermo Fisher Scientific) [39], but it is still a challenge for large-scale WGS assays. In order to achieve this goal concerning big data in the near future, new bioinformatics tools must be developed, or the current ones further improved.

Willems et al. [26] analyzed human STR variation by using lobSTR. These authors employed the data of Phase 1 of the 1000 Genomes Project. The data were generated by using low-sequencing coverage, which is excessively error-prone. In fact, the authors reported difficulties in detecting both alleles in each sample, which resulted in an overall deficit of heterozygotes. As previously addressed, several reasons led us to choose HipSTR to call STR genotypes from this high-coverage dataset of the 1000 Genomes Project. Because HipSTR allows the flanking regions to be customized, almost any STR marker can be evaluated in hundreds of samples at once. At first glance, HipSTR may appear more complex, but it is the most appropriate tool to deal with whole genomes. In addition, a recent evaluation of the performance of this tool revealed high efficiency and accuracy levels [14].

Although HipSTR provides flexibility, the major limitation of this study is the inability to genotype D21S11, which is one of the 20 CODIS loci. Additional limitations are the failure in detecting two very small Penta D alleles and the biased allele frequencies of very large Penta D and Penta E alleles probably because of sequence-specific features, such as the GC content [40,41,42] producing low depth of coverage bias and/or the limited length of the Illumina NGS reads (150 bp paired-end reads). This issue could be immediately circumvented with long-read sequencing technologies, such as those implemented in Pacific Biosciences (PacBio) and Oxford Nanopore platforms. However, one should not expect that long-read sequencing would be suitable for a wide range of forensic samples, which are often degraded and/or available in low amounts [40,41,42,43]. It is noteworthy that, by employing 300 nucleotide-long paired-end reads in a targeted sequencing assay, we successfully genotyped D21S11 with HipSTR, which suggests a sequencing methodology issue rather than a bioinformatics issue [14].

In this study, Penta D and Penta E showed 10.74% and 15.81% of missing data, respectively. By using Illumina sequencing technology, van der Gaag et al. [37] showed that longer alleles of Penta D, Penta E, and FGA presented sequencing errors at the end of the reads, which resulted in null alleles and genotyping errors. As observed for D21S11, this issue was probably related to the impossibility of detecting longer alleles due to read-length constraints. Furthermore, we did not detect two very small Penta D alleles (2.2 and 3.2), which are common in African populations, which was unexpected. Supplementary Table S4 compares the allele frequencies estimated in the present study with the allele frequencies obtained from the SPSmart STR browser (PopSTR) [24] for the major population groups. Such straightforward comparison showed that we were not able to detect alleles larger than 18 in Penta E. This failure led directly to Hardy-Weinberg equilibrium deviations (Table S3) due to deficit of heterozygotes in 24 out of the 26 studied populations. Thus, allele frequencies estimated for Penta E were strongly biased toward increased frequencies of shorter alleles and have limited applicability (Supplementary Table S2). The probabilities obtained with the *F_ST_* analysis (Table 4) supported this conclusion: Penta E presented significant *F_ST_* values in all five comparisons. Although Penta D and FGA also posed this problem, their undetected alleles usually have low frequencies—Except for Penta D alleles 2.2 and 3.2 in African populations (Supplementary Table S4). Therefore, this technical issue did not influence the Hardy-Weinberg equilibrium and *F_ST_* analysis as much as Penta E. Although this comparison is valid and helpful, we must emphasize that the compared samples corresponded to distinct population groups. The African population group in popSTR comprised mainly East African Somalian individuals (404 out of 507 samples), while the African populations in the 1000 Genomes Project samples corresponded to West Africa. Similarly, over 50% of the European population group in popSTR was composed mainly of U.S. Europeans (1443 out of 2135) [5,24]. Taken together, these results attest that the bioinformatics analysis performed in the present study is robust, and that the distribution of allele frequencies is reliable for all loci except Penta E.

The most polymorphic loci in the whole dataset of the 1000 Genomes Project were D1S1656, D2S1338, D12S391, D18S51, and FGA. All these markers presented high degrees of polymorphism throughout the world. AMOVA revealed that most of the variance (97.12%) in allele frequencies occurred within populations, corroborating previous studies [44,45]. A study that evaluated human population structure using genotypes at 377 autosomal microsatellite loci in 1056 individuals from 52 worldwide populations revealed that the variance within populations accounts for 93 to 95% of genetic variation, while differences among major groups constitute only 3 to 5% [45,46]. Although the number of populations and genetic markers are quite different, the larger amount of variance within populations and lower variance among groups observed in the present study may be either due to chance or to the fact that forensic STRs do show relatively lower *F_ST_* than random STRs due to the increased heterozygosity of the former [46]. However, as expected, AMOVA, together with principal component analysis (Figure 1) and the clustering analysis performed with STRUCTURE (Figure 2), confirmed that the four ancestral populations groups (AFR, EUR, EAS, and SAS) defined by the 1000 Genomes Consortium did differ significantly from each other. Given that the admixed American populations present different ancestry compositions (Figure 2), most of them clustered with Europeans, while ACB and ASW clustered with Africans (Figure 1).

The results obtained with the STRUCTURE software corroborated the relationship between the different population groups and provided additional support for the reliability of the calculated genotypes. When *k* = 3, SAS resembled an admixture between EAS and EUR. A specific cluster for SAS emerged when *k* = 4. When *k* = 5, a minor Eurasian (shared between EUR and EAS) component arose. When *k* = 6, the SAS-shared ancestry with EUR and EAS became more evident. Regarding the admixed American populations, irrespective of the number of clusters considered, ACB and ASW revealed their preeminent African origin, CLM and PUR revealed more extensive European ancestry, and MXL and PEL revealed almost equal amounts of European and Amerindian (i.e., EAS) ancestries. These results fully corroborated the distribution of the populations into the PCoA (Figure 1). Additional clusters did not provide increased resolution with straightforward meaning.

The outcome of this population genetics evaluation further corroborates the robustness and reliability of this STR dataset. Despite all the applications already addressed in the beginning of this section, the most important contribution of this open access genotype dataset probably lies in the fact that it may be used to estimate and establish additional population genetics parameters that may be taken as direct references in many studies that are using the 1000 Genomes Project dataset to retrieve new sets of SNPs, indels and microhaplotypes in various efforts to maximize intelligence from DNA evidence [27,47,48,49,50].

## 5. Conclusions

We were able to offer a reliable open-access STR database based on the high-coverage (30x) WGS data of Phase 3 of the 1000 Genomes Project generated by the NYGC. However, the limited length of sequencing reads introduces noticeable bias in allele frequencies estimated for Penta D and Penta E. The reliability of this dataset is supported by (a) previous studies attesting that HipSTR is efficient, (b) the Hardy-Weinberg equilibrium analysis, (c) the set of analyses employed to evaluate the interpopulation genetic diversity, and (d) the comparison between the allele frequencies obtained here and the frequencies obtained by other initiatives that used capillary electrophoresis. Although we expect that this open-access database will be of great interest for future forensic studies on population genetics, the current 1000 Genomes Project dataset does not describe human genetic diversity worldwide. In fact, many biogeographical regions, mainly in Oceania and the Americas, have not been sampled, indicating that additional large-scale initiatives may provide further insight into STR diversity in populations worldwide.

## Figures and Tables

**Table 1:** Average coverages obtained for each STR marker using the HipSTR tool.

Marker	Lowest Value	Median	Highest Value	Mean	Standard Deviation
CSF1PO	21	44	91	44.54	8.29
D1S1656	24	45	92	45.48	8.58
D2S441	26	49	131	49.17	9.04
D2S1338	28	50	105	51.28	9.64
D3S1358	28	51	119	51.73	9.33
D5S818	20	42	98	42.90	8.14
D7S820	20	38	86	39.00	7.78
D8S1179	26	47	96	48.09	8.95
D10S1248	18	40	100	40.75	7.99
D12S391	26	52	113	52.53	9.47
D13S317	11	37	79	37.93	7.50
D16S539	21	44	92	44.70	8.49
D18S51	24	47	91	47.38	9.07
D19S433	19	45	89	45.28	8.62
D22S1045	22	49	111	49.47	9.11
FGA	23	50	118	51.33	9.49
Penta D	19	43	95	43.71	8.74
Penta E	18	41	107	41.49	8.04
TH01	16	40	83	40.73	8.01
TPOX	15	37	86	37.14	7.66
vWA	21	47	105	48.44	9.36

**Table 2:** Allelic frequencies and the forensic parameters estimated for each STR marker in the whole dataset of the 1000 Genomes Project.

Allele	CSF1PO	D1S1656	D2S441	D2S1338	D3S1358	D5S818	D7S820	D8S1179	D10S1248	D12S391	D13S317	D16S539	D18S51	D19S433	D22S1045	FGA	Penta D	Penta E	TH01	TPOX	vWA
5											0.0002						0.0125	0.0861	0.0020		
6							0.0004		0.0002					0.0004			0.0034	0.0017	0.1905	0.0210	
7	0.0198	0.0002				0.0118	0.0178		0.0002		0.0018	0.0004		0.0002			0.0150	0.1084	0.2828	0.0074	
7.3	0.0002																				
8	0.0218	0.0080	0.0006	0.0002		0.0222	0.1842	0.0064	0.0006		0.1394	0.0314		0.0002	0.0006		0.0539	0.0854	0.1287	0.4217	
8.3																			0.0002		
9	0.0334	0.0004	0.0018		0.0004	0.0421	0.0942	0.0066	0.0004		0.0891	0.1805	0.0004	0.0012	0.0002		0.2195	0.0358	0.2314	0.1534	
9.2			0.0052				0.0016													0.0002	
9.3																			0.1491		
10	0.2398	0.0080	0.2264			0.0976	0.2534	0.0953	0.0010		0.0763	0.1123	0.0046	0.0028	0.0154		0.1660	0.0873	0.0150	0.0621	0.0004
10.2			0.0004				0.0004						0.0006								
10.3	0.0002																		0.0002		
11	0.2658	0.0788	0.3401		0.0004	0.3187	0.2506	0.0753	0.0166		0.2650	0.2888	0.0110	0.0210	0.1649		0.1839	0.1774	0.0002	0.2971	0.0014
11.2							0.0004				0.0002			0.0008							
11.3			0.0529																		
11.4																	0.0002				
12	0.3375	0.0732	0.1062		0.0022	0.3097	0.1650	0.1180	0.0684		0.2912	0.2336	0.0805	0.0745	0.0170		0.1506	0.1886		0.0359	0.0008
12.2							0.0002						0.0006	0.0122							
12.3			0.0022											0.0004							
13	0.0693	0.1142	0.0282	0.0002	0.0034	0.1841	0.0275	0.2340	0.2616		0.0982	0.1317	0.1155	0.2706	0.0034		0.1380	0.1065		0.0006	0.0072
13.2													0.0032	0.0341							
13.3			0.0008						0.0002				0.0002								
14	0.0108	0.1472	0.2121	0.0006	0.0777	0.0116	0.0042	0.2418	0.2816	0.0010	0.0370	0.0200	0.1674	0.2726	0.0509		0.0396	0.0669		0.0006	0.1255
14.2													0.0010	0.0623			0.0002				0.0004
14.3		0.0028	0.0004										0.0002								
15	0.0016	0.1772	0.0202	0.0014	0.3075	0.0020		0.1566	0.2220	0.0336	0.0016	0.0014	0.1670	0.1042	0.3313	0.0004	0.0134	0.0380			0.1217
15.1														0.0002							
15.2					0.0006								0.0004	0.0799							0.0002
15.3		0.0272							0.0002												
16		0.1410	0.0026	0.0318	0.3031	0.0002		0.0559	0.1178	0.0342			0.1414	0.0295	0.2502	0.0006	0.0031	0.0176			0.2251
16.2					0.0002					0.0004				0.0240		0.0004					
16.3		0.0560											0.0002								
17		0.0448		0.1328	0.2063			0.0080	0.0268	0.1109			0.1187	0.0046	0.1472	0.0016	0.0007	0.0005			0.2433
17.2		0.0004								0.0014				0.0034							
17.3		0.0796								0.0078											
18		0.0060		0.0897	0.0903			0.0022	0.0022	0.2264			0.0787		0.0170	0.0110					0.1753
18.2										0.0008			0.0002	0.0008		0.0030					
18.3		0.0298								0.0096						0.0002					0.0002
19		0.0006		0.1679	0.0072				0.0002	0.1743			0.0519		0.0014	0.0673					0.0770
19.2										0.0030						0.0016					
19.3		0.0040								0.0044											
20				0.1106	0.0008					0.1415			0.0308		0.0004	0.0906					0.0206
20.2										0.0004			0.0002			0.0012					
20.3		0.0006								0.0002											
21				0.0637						0.0911			0.0130		0.0002	0.1247					0.0006
21.2																0.0034					
22				0.0813						0.0741			0.0076			0.1785					0.0004
22.2																0.0046					
23				0.1306						0.0552			0.0028			0.1679					
23.2																0.0034					
23.3																0.0002					
24				0.1012						0.0180			0.0014			0.1619					
24.2																0.0048					
25				0.0685						0.0104			0.0004			0.1026					
25.2																0.0028					
25.3																0.0002					
26				0.0154						0.0014						0.0436					
26.2																0.0012					
26.3																0.0002					
27				0.0030												0.0137					
27.2																0.0002					
28				0.0010												0.0054					
29				0.0002												0.0026					
30																0.0002					
N	2504	2500	2504	2504	2504	2504	2494	2504	2500	2502	2485	2502	2497	2496	2504	2490	2235	2108	2502	2504	2499
Na	11	21	15	18	13	10	13	11	16	22	11	9	27	22	14	31	15	13	10	10	16
Ho	0.7492	0.8440	0.7380	0.8722	0.7496	0.7220	0.7927	0.8103	0.7536	0.8437	0.7666	0.7838	0.8614	0.8121	0.7364	0.8305	0.7808	0.4877	0.7450	0.6621	0.7943
He	0.7512	0.8893	0.7729	0.8902	0.7569	0.7567	0.8020	0.8305	0.7836	0.8665	0.8010	0.7983	0.8801	0.8227	0.7755	0.8728	0.8439	0.8803	0.7914	0.7048	0.8226
MP	0.1059	0.0224	0.0833	0.0226	0.1002	0.0978	0.0690	0.0499	0.0785	0.0324	0.0676	0.0697	0.0267	0.0509	0.0841	0.0287	0.0423	0.0420	0.0737	0.1341	0.0548
PE	0.5084	0.6831	0.4895	0.7391	0.5091	0.4632	0.5856	0.6183	0.5159	0.6825	0.5386	0.5693	0.7175	0.6217	0.4868	0.6569	0.5638	0.1769	0.5013	0.3722	0.5885
PD	0.8941	0.9776	0.9167	0.9774	0.8998	0.9022	0.9310	0.9501	0.9215	0.9676	0.9324	0.9303	0.9733	0.9491	0.9159	0.9713	0.9577	0.9580	0.9263	0.8659	0.9452
PIC	0.7104	0.8790	0.7393	0.8797	0.7169	0.7180	0.7726	0.8088	0.7501	0.8526	0.7738	0.7688	0.8680	0.8021	0.7421	0.8593	0.8244	0.8684	0.7589	0.6575	0.7985

N: number of samples; Na: number of alleles; Ho: observed heterozygosity; He: expected heterozygosity; MP: match probability; PE: power of exclusion; PD: power of discrimination; PIC: polymorphism information content.

**Table 3:** Probabilities of adherence to Hardy-Weinberg equilibrium proportions for each STR in all the 26 subpopulations analyzed in the 1000 Genomes Project. Significant *p*-values (α = 0.05) are in boldface. The probabilities that remained significant after the Bonferroni correction for multiple tests (αBONFERRONI = 0.05/546 = 0.000092) are also underlined.

POP	CSF1PO	D1S1656	D2S441	D2S1338	D3S1358	D5S818	D7S820	D8S1179	D10S1248	D12S391	D13S317	D16S539	D18S51	D19S433	D22S1045	FGA	Penta D	Penta E	TH01	TPOX	vWA
**ACB**	0.8317	0.1003	0.9562	0.6768	0.7289	0.5439	**0.0238**	0.1626	0.7625	0.9937	0.1509	0.9058	0.1797	0.9795	0.8294	0.8814	0.1985	0.2895	0.4184	0.0678	0.1618
**ASW**	0.8494	0.8119	0.9805	0.7607	0.8298	0.8945	0.6251	0.9977	0.5225	0.4894	0.2447	0.9927	0.6351	0.3402	0.4035	0.8225	0.3519	0.0724	0.9511	0.9248	0.9409
**BEB**	0.6165	0.6321	0.1621	**0.0216**	0.9740	0.6614	0.4515	0.9476	0.8795	0.9124	0.5829	0.6662	0.3397	0.8489	0.6579	0.8494	0.4690	**0.0000**	0.2040	0.4850	0.9571
**CDX**	0.9917	0.3823	0.4668	0.9308	0.4053	**0.0000**	0.5757	0.9797	0.5425	0.4254	0.4678	0.2605	0.6701	0.5218	0.2454	0.1603	0.4560	**0.0000**	**0.0268**	**0.0025**	0.4988
**CEU**	0.9331	0.5301	0.9948	0.1233	0.1851	0.0674	0.6688	0.2600	0.8395	0.5314	0.8354	0.9559	0.1012	0.9471	0.8354	0.8997	0.5208	**0.0000**	0.7591	0.4191	0.1209
**CHB**	0.3105	0.7945	**0.0005**	0.8305	0.9407	0.8190	0.4159	0.2194	0.7585	**0.0003**	0.9664	0.4847	0.1581	**0.0047**	0.9689	0.9747	**0.0011**	**0.0000**	0.9424	0.8976	**0.0000**
**CHS**	0.2302	0.1077	0.2844	0.6237	0.5740	0.0727	0.9894	0.4883	0.3859	0.6180	0.7626	0.6386	0.3969	**0.0391**	0.8843	0.9618	0.4278	**0.0000**	0.1768	0.7723	0.8666
**CLM**	0.9075	0.3108	0.4415	0.0684	0.3560	**0.0470**	**0.0000**	0.8558	0.5450	**0.0004**	0.1412	0.5892	0.0829	0.9682	0.9999	0.9976	0.6915	**0.0000**	0.4287	0.4076	0.7427
**ESN**	0.1175	0.9988	0.9706	0.9750	**0.0303**	0.2028	0.6773	**0.0131**	0.8721	0.1069	0.8443	0.9823	0.9529	0.4869	**0.0209**	1.0000	**0.0110**	**0.0000**	0.9238	0.3436	0.0579
**FIN**	0.9558	0.4612	0.7627	**0.0000**	0.5922	0.5269	0.8596	0.3818	0.8976	0.9531	0.9688	0.7919	0.2147	0.9665	0.9869	0.7378	0.9930	**0.0000**	0.9504	0.8369	0.9465
**GBR**	0.9311	0.9506	0.2788	0.8505	0.9925	0.8037	0.8379	0.9828	0.8791	0.8061	0.2196	**0.0259**	0.2483	**0.0000**	**0.0317**	0.9879	0.2263	**0.0000**	0.0512	0.4530	0.4718
**GIH**	0.6965	**0.0239**	0.8370	0.6288	0.9993	0.4325	0.6899	**0.0011**	0.8836	0.9808	0.3863	0.6818	0.9979	0.7126	0.4995	0.7371	0.6790	**0.0000**	0.0770	0.7827	0.3344
**GWD**	0.1950	0.6832	0.9970	0.9978	0.5107	0.8942	0.2703	0.3213	0.9718	0.9527	0.4987	0.7810	**0.0098**	0.9973	0.2150	0.1932	**0.0000**	**0.0000**	0.1804	0.8530	0.9779
**IBS**	**0.0000**	0.8111	0.9373	0.9690	0.1246	0.9874	0.8882	0.8501	0.2448	0.5344	0.9012	0.1202	0.9943	0.9706	0.9373	0.5506	**0.0333**	**0.0000**	0.4393	0.6766	0.3111
**ITU**	0.6156	0.3367	**0.0097**	0.7459	0.7367	0.9723	0.8685	0.8721	0.1101	0.9617	0.7853	0.5636	0.9777	0.9016	0.9803	**0.0404**	0.8992	**0.0000**	0.9027	0.3481	0.4022
**JPT**	0.8554	0.7945	0.5003	0.7191	0.6566	0.6312	0.7590	0.2612	0.8154	0.7891	0.3862	0.9589	0.9922	0.9952	**0.0006**	0.7052	0.9159	**0.0000**	0.7771	**0.0002**	0.8666
**KWV**	0.9942	**0.0025**	0.8299	0.9795	0.9899	0.2980	0.2296	0.3737	0.7030	0.9483	0.5815	0.9489	0.1698	0.5107	0.9073	0.1862	0.0000	**0.0000**	0.5244	**0.0006**	0.4226
**LWK**	0.3621	0.9913	0.9838	0.3363	0.5245	0.8976	0.6751	0.6144	0.8436	0.1478	0.6934	0.7081	0.2947	0.4416	0.9396	0.9998	0.6011	**0.0000**	0.9548	**0.0325**	0.9572
**MSL**	0.8099	0.2244	0.4496	0.7258	0.9316	0.0628	**0.0372**	0.7088	0.0992	0.9442	0.9897	0.8470	1.0000	0.9398	0.2779	0.1057	0.2086	**0.0000**	**0.0171**	0.5908	**0.0000**
**MXL**	**0.0047**	**0.0109**	0.8297	0.6202	0.1254	0.8631	0.5456	**0.0125**	0.0509	0.4395	0.8989	0.7887	**0.0000**	0.3860	0.9233	0.1562	0.5281	**0.0000**	0.3747	0.4029	0.5094
**PEL**	0.8460	0.1247	0.9976	0.7122	0.1681	0.9781	0.7676	0.7192	0.9635	0.9402	0.1281	0.6186	0.9833	0.9176	0.8786	**0.0000**	0.5730	**0.0000**	0.8399	0.2170	**0.0467**
**PJL**	0.8683	**0.0073**	1.0000	0.6486	0.8966	**0.0001**	0.8996	0.5188	0.8722	0.5388	0.9943	0.4565	0.9976	0.6490	0.9166	**0.0053**	0.1485	**0.0000**	0.0721	0.6604	0.2985
**PUR**	0.7847	0.0819	**0.0058**	0.9097	0.1398	0.8342	0.7698	0.4704	**0.0034**	**0.0045**	**0.0337**	0.0556	0.7141	**0.0006**	0.9810	0.7965	0.7547	**0.0000**	**0.0000**	0.2571	**0.0191**
**STU**	0.9028	0.5930	**0.0000**	0.8290	0.2546	0.2661	**0.0071**	0.4770	0.9882	0.2049	0.0952	0.1927	0.4262	0.2335	**0.0001**	0.6382	0.2129	**0.0000**	0.6082	0.5311	0.9627
**TSI**	0.6299	0.4393	0.9777	0.0805	0.5719	0.4424	0.6240	0.4107	**0.0356**	0.6573	0.5777	0.9581	**0.0074**	**0.0000**	0.1240	0.5698	0.3556	**0.0000**	**0.0203**	0.3932	0.8569
**YIR**	0.6908	0.2395	0.8925	0.3004	**0.0000**	0.8611	0.7701	0.9277	0.6050	0.3079	0.5197	0.4061	0.9867	0.8417	0.9451	0.9643	0.4923	**0.0000**	0.4285	0.1299	0.9942

**Table 4:** *F_ST_* and probabilities of population non-differentiation comparing population groups of the 1000 Genomes Project to those of the SPSmart STR browser (PopSTR) for each STR marker. Significant *p*-values (α = 0.05) are in boldface. The probabilities that remained significant after the Bonferroni correction for multiple tests (αBONFERRONI = 0.05/105 = 0.00048) are also underlined.

Marker	AFR		AMR		EAS		EUR		SAS	
	*F_ST_*	*p*-value	*F_ST_*	*p*-Value	*F_ST_*	*p*-Value	*F_ST_*	*p*-Value	*F_ST_*	*p*-Value
**CSF1PO**	0.0084	**0.0057 ± 0.0007**	−0.00104	0.7571 ± 0.0047	−0.0014	0.6656 ± 0.0053	0.0004	0.2445 ± 0.0044	0.0019	0.1951 ± 0.0036
**D1S1656**	0.0024	**0.0355 ± 0.0019**	0.00028	0.3149 ± 0.0046	−0.0024	0.9774 ± 0.0015	0.0045	**0.0000 ± 0.0000**	0.0021	0.1152 ± 0.0032
**D2S441**	0.0093	**0.0005 ± 0.0002**	0.00370	**0.0216 ± 0.0015**	0.0025	0.1251 ± 0.0027	0.0074	**0.0000 ± 0.0000**	0.0122	**0.0058 ± 0.0007**
**D2S1338**	0.0124	**0.0000 ± 0.0000**	−0.00048	0.6527 ± 0.0049	−0.0026	0.9940 ± 0.0007	0.0031	**0.0008 ± 0.0003**	0.0024	0.1044 ± 0.0030
**D3S1358**	0.0044	**0.021 ± 0.0014**	−0.00122	0.8369 ± 0.0040	0.0010	0.2578 ± 0.0044	0.0013	0.0835 ± 0.0026	0.0033	0.1143 ± 0.0031
**D5S818**	0.0030	**0.0477 ± 0.0024**	0.00609	**0.0051 ± 0.0008**	−0.0014	0.6936 ± 0.0048	−0.0007	0.7638 ± 0.0047	−0.0014	0.6250 ± 0.0050
**D7S820**	0.0047	**0.0095 ± 0.0009**	−0.00105	0.7982 ± 0.0041	−0.0006	0.5151 ± 0.0050	−0.0003	0.5677 ± 0.0054	−0.0023	0.8731 ± 0.0034
**D8S1179**	0.0033	**0.0308 ± 0.0018**	−0.00158	0.9817 ± 0.0014	−0.0016	0.7986 ± 0.0042	0.0024	**0.0150 ± 0.0012**	−0.0003	0.4615 ± 0.0049
**D10S1248**	0.0017	0.1081 ± 0.0033	−0.00132	0.8984 ± 0.0029	−0.0031	0.9976 ± 0.0005	0.0003	0.2627 ± 0.0044	−0.0001	0.4055 ± 0.0046
**D12S391**	0.0007	0.1920 ± 0.0041	0.00077	0.1805 ± 0.0036	−0.0019	0.8774 ± 0.0030	0.0008	0.0946 ± 0.0030	−0.0009	0.6155 ± 0.0047
**D13S317**	0.0160	**0.0000 ± 0.0000**	0.00017	0.3478 ± 0.0044	−0.0018	0.7993 ± 0.0045	0.0003	0.2904 ± 0.0047	0.0051	**0.0354 ± 0.0019**
**D16S539**	0.0105	**0.0002 ± 0.0001**	−0.00051	0.5773 ± 0.0047	−0.0010	0.5939 ± 0.0046	0.0047	**0.0009 ± 0.0003**	−0.0010	0.5987 ± 0.0052
**D18S51**	0.0018	**0.0483 ± 0.0019**	−0.00138	0.9831 ± 0.0013	−0.0007	0.5795 ± 0.0055	0.0005	0.2028 ± 0.0042	−0.0003	0.4671 ± 0.0047
**D19S433**	0.0093	**0.0003 ± 0.0002**	0.00151	0.1085 ± 0.0027	0.0010	0.2476 ± 0.0041	0.0000	0.3759 ± 0.0049	0.0036	0.0660 ± 0.0028
**D22S1045**	0.0004	0.2943 ± 0.0053	0.00217	0.0766 ± 0.0025	0.0094	**0.0055 ± 0.0007**	0.0034	**0.0087 ± 0.0009**	0.0054	0.0516 ± 0.0021
**FGA**	0.0011	0.1503 ± 0.0037	−0.00089	0.8382 ± 0.0034	0.0036	**0.0397 ± 0.0020**	0.0007	0.1306 ± 0.0035	0.0018	0.1518 ± 0.0039
**Penta D**	0.0275	**0.0000 ± 0.0000**	0.00318	**0.0113 ± 0.0010**	−0.0015	0.7660 ± 0.0042	0.0003	0.2969 ± 0.0042	0.0000	0.4117 ± 0.0048
**Penta E**	0.0139	**0.0000 ± 0.0000**	0.00733	**0.0000 ± 0.0000**	0.0412	**0.0000 ± 0.0000**	0.0083	**0.0000 ± 0.0000**	0.0179	**0.0000 ± 0.0000**
**TH01**	0.0103	**0.0004 ± 0.0002**	0.00090	0.2083 ± 0.0036	−0.0025	0.9262 ± 0.0027	0.0057	**0.0001 ± 0.0001**	0.0026	0.1425 ± 0.0038
**TPOX**	0.0039	**0.0269 ± 0.0019**	−0.00057	0.5622 ± 0.005	−0.0011	0.5341 ± 0.0046	0.0015	0.0939 ± 0.0033	0.0063	**0.0455 ± 0.0020**
**vWA**	0.0065	**0.0007 ± 0.0003**	−0.00095	0.7575 ± 0.0047	0.0005	0.3135 ± 0.0047	−0.0001	0.4312 ± 0.0051	−0.0010	0.5977 ± 0.0049

## Data Availability

1000 Genomes Project Phase 3 samples in a high-coverage (30x) assay using the NovaSeq 6000 Sequencing System (Illumina, Inc.) https://www.internationalgenome.org/data-portal/data-collection/30x-grch38 accessed on 10 July 2021.

## Supplementary Materials

The following supporting information can be downloaded at: https://www.mdpi.com/article/10.3390/genes13122205/s1. Table S1: ST1-STR genotypes defined using HipSTR from the high-coverage New York Genome Center (NYGC) dataset released by the 1000 Genomes Project. Table S2: Summary of information necessary to understand the ST2 set of tables. Table S3: Summary of information necessary to understand the ST3 set of tables. Table S4: Summary of information necessary to understand the ST4 set of tables.
